# Alkaline-Tolerant *Bacillus cereus* 12GS: A Promising Polyhydroxybutyrate (PHB) Producer Isolated from the North of Mexico

**DOI:** 10.3390/microorganisms12050863

**Published:** 2024-04-26

**Authors:** Gustavo de J. San Miguel-González, María E. Alemán-Huerta, Raul E. Martínez-Herrera, Isela Quintero-Zapata, Susana de la Torre-Zavala, Hamlet Avilés-Arnaut, Fátima L. Gandarilla-Pacheco, Erick de J. de Luna-Santillana

**Affiliations:** 1 Instituto de Biotecnología, Facultad de Ciencias Biológicas, Universidad Autónoma de Nuevo León, Av. Pedro de Alba y Manuel L. Barragán S/N, San Nicolás de los Garza C.P. 66455, Nuevo León, Mexico; gustavo.sanmiguelg@uanl.edu.mx (G.d.J.S.M.-G.); isela.quinterozp@uanl.edu.mx (I.Q.-Z.); susana.delatorrezv@uanl.edu.mx (S.d.l.T.-Z.); hamlet.avilesarn@uanl.edu.mx (H.A.-A.); fatima.gandarillap@uanl.edu.mx (F.L.G.-P.); 2 Escuela de Ingenería y Ciencias, Tecnológico de Monterrey, Av. Eugenio Garza Sada 2501 Sur, Tecnológico, Monterrey C.P. 64849, Nuevo León, Mexico; 3 Institute of Advanced Materials for Sustainable Manufacturing, Tecnológico de Monterrey, Av. Eugenio Garza Sada 2501 Sur, Tecnológico, Monterrey C.P. 64849, Nuevo León, Mexico; 4 Laboratorio Medicina de Conservación, Centro de Biotecnología Genómica, Instituto Politécnico Nacional, Blvd. del Maestro esq, Elías Piña, Colonia Narciso Mendoza, Reynosa C.P. 88700, Tamaulipas, Mexico; edeluna@ipn.mx

**Keywords:** *Bacillus cereus*, fermentations, isolation, polyhydroxybutyrate, Taguchi design

## Abstract

Environmental pollution caused by petroleum-derived plastics continues to increase annually. Consequently, current research is interested in the search for eco-friendly bacterial polymers. The importance of *Bacillus* bacteria as producers of polyhydroxyalkanoates (PHAs) has been recognized because of their physiological and genetic qualities. In this study, twenty strains of *Bacillus* genus PHA producers were isolated. Production was initially evaluated qualitatively to screen the strains, and subsequently, the strain B12 or *Bacillus* sp. 12GS, with the highest production, was selected through liquid fermentation. Biochemical and molecular identification revealed it as a novel isolate of *Bacillus cereus*. Production optimization was carried out using the Taguchi methodology, determining the optimal parameters as 30 °C, pH 8, 150 rpm, and 4% inoculum, resulting in 87% and 1.91 g/L of polyhydroxybutyrate (PHB). Kinetic studies demonstrated a higher production within 48 h. The produced biopolymer was analyzed using Fourier-transform infrared spectroscopy (FTIR), confirming the production of short-chain-length (scl) polyhydroxyalkanoate, named PHB, and differential scanning calorimetry (DSC) analysis revealed thermal properties, making it a promising material for various applications. The novel *B. cereus* isolate exhibited a high %PHB, emphasizing the importance of bioprospecting, study, and characterization for strains with biotechnological potential.

## 1. Introduction

In the modern era, plastics have become an essential component of our everyday existence, consequently resulting in a substantial increase in their production, surging from 1.5 million metric tons in 1950 to roughly 370 million metric tons in recent years [1]. In response to this issue, heightened endeavors are being directed towards the creation of biodegradable, bio-based polymers derived from sustainable resources, aimed at mitigating the accumulation of synthetic plastics [2]. Intensive research has recently focused on developing alternatives to synthetic plastics, notably biodegradable polymers and polymers synthesized through microbial processes [3].

Polyhydroxyalkanoates (PHAs), one of the representative natural polyester-based biodegradable polymers, are a family of thermoplastic polymers that are produced by several microorganisms growing under stress conditions, mostly by excessive carbon sources and nitrogen limitations. The PHA family is considered biodegradable, non-toxic, eco-friendly, and can be produced from sustainable resource materials [4]. The monomer units of PHA can be categorized depending on their carbon atom count: short-chain- and medium-chain-length PHAs (scl-PHA and mcl-PHA). Scl-PHA materials have thermoplastic properties like those of synthetic plastics. One of the polymer’s scl-PHA representatives is polyhydroxybutyrate (PHB), due to it containing a methyl group in its chemical structure; additionally, PHB has a considerably higher crystallinity, enhanced thermal stability, and reduced oxygen permeability compared to other members [5,6]. PHB or PH3B has gained much attention as a replacement for non-biodegradable commercial polymers due to mechanical strength attributes analogous to those of polyethylene (PE) and polypropylene (PP). PHB exhibits full biodegradability under diverse natural dynamic settings, including soil, industrial composting, and seawater, by the presence of diverse aerobic and anaerobic microbial consortia [7,8]. Moreover, these inherent physical properties of PHB enable its diverse application in various commercial scenarios, such as its utilization as a packaging material, agricultural coating, and as a carrier in drug delivery systems [9].

Consequently, there is an increasing demand for PHB production. According to statistics from the Nova Institute, global PHB production was projected to increase from 34 thousand metric tons in 2013 to approximately 7.4 thousand metric tons in 2018. However, the significant obstacle hindering large-scale PHB production remains its production cost [10]. Considerable endeavors have been invested in mitigating the production expenses of PHB in recent years. This has been achieved by searching for efficient bacterial strains and optimizing both the fermentation and recovery procedures [11].

Although the accumulation of these carbonosomes has been investigated in various microorganisms, *Bacillus* species have shown a yield of up to 90% PHA from the total dry cells produced by liquid fermentation. Because its physiological versatility enables it to widely distributed in nature, *Bacillus* spp. is found in almost all habitats, from extreme to temperate climates throughout the world. This genus is suggested as a viable candidate among microorganisms for PHA production, primarily due to its capacity to attain elevated yields, while requiring a minimal number of fermentation factors [7,12]. The cell size of the *Bacillus* ranges from 0.6 to 1.2 μm in width and 0.9–8 μm in length [13]. However, the size may vary depending on the species, culture medium, and growth conditions. On the other hand, agricultural soil has been differentiated from other soils by reporting higher numbers of PHB-producing isolates [14].

The objective of the present study was to isolate bacteria of genus *Bacillus* producers’ microbial biopolymers, screening strains by qualitative techniques, and select a hyper-producing strain using the quantitative technique (liquid fermentation) for PHB production. This study also aimed to determine the identity of the strain through molecular techniques. In addition, we use a Taguchi experimental design to determine the optimal fermentation parameters, and probe with chemical analysis the structure of PHB, as well as its thermal stability.

## 2. Materials and Methods

### 2.1. Chemicals and Substrates

All analytical-grade chemicals and substrates used in this project were obtained from Sigma-Aldrich Co. (St. Louis, MO, USA).

### 2.2. Sample Collection and Isolation of Bacterial Strains

Soil samples were collected from agricultural soil in General Teran, Nuevo León, Mexico (25°20′45.0″ N 99°35′29.0″ W) and subjected to treatment for the isolation of microbial PHB producers. For this, 1 g of soil sample was dissolved in 10 mL of sterile distilled water and heated at 80 °C for 10 min, to isolate only endospore-forming bacteria (*Bacillus*). Serial dilutions of this sample were carried out up to 10^−5^. Then, 1 mL of the diluted sample was poured onto nutritive agar plates and incubated at 30 °C for 48 h [15]. A total of 20 bacterial colonies were isolated and underwent staining studies (simple staining, Gram staining, and spore staining) to confirm their morphology, Gram status, and spore presence.

### 2.3. Qualitative Screening of PHB-Producer Bacterial Strains

#### 2.3.1. Sudan Black B Staining

A thin smear of isolated bacteria was prepared on clean slides and heat-fixed. The smeared surface was then stained with Sudan black B solution (0.3% *w*/*v*) for 10 min. Afterward, it was washed with xylene and stained with safranin (0.5% *w*/*v*) for 5 min. Following this, the stained smear was washed with distilled water and dried. The smear was observed under a BA410E light microscope (Motic, Hong Kong) at a magnification of 100× [16].

#### 2.3.2. Nile Blue A Staining

The isolates were smeared and heat-fixed on clean slides, stained with Nile blue A dye (1% *w*/*v*) at 55 °C for 10 min, and washed with an acetic acid solution (8% *v*/*v*) and then with distilled water. Finally, the preparation was covered with a clean coverslip and visualized using a VE-146YT fluorescence microscope (VELAB tm, Pharr, TX, USA) at a magnification of 100× with a wavelength of 460 nm. This procedure was carried out to detect intracellular PHB granules, which appear as orange fluorescence [17]. The most efficient PHB producers among the bacterial isolates were identified through the analysis of Nile blue staining intensity by ImageJ V. 1.53. The integrated density of PHB-accumulating cells in the isolates was determined by analyzing Nile blue-stained images obtained from fluorescent microscopy studies using the ImageJ program plugin [18].

### 2.4. Quantitative Screening of PHB-Producer Bacterial Strains

#### 2.4.1. Evaluation of Biomass Generation and PHB Production

The biomass generation and PHB production of qualitatively selected bacterial strains were assessed using GRPD growth medium at pH 6. This medium comprises glucose (15 g/L), peptone (2 g/L), yeast extract (2.5 g/L), and NaCl (1.25 g/L) [19]. To conduct these assessments, we followed the methodology outlined by Martínez-Herrera et al. [20]. In this method, a 250 mL flask pre-inoculum, containing 100 mL of GRPD growth medium, was inoculated with 100 μL (10^8^ colony-forming units [CFU]/mL) of a spore solution of the selected bacterial strains and incubated at 30 °C and 150 rpm for 24 h in a MaxQ4000 shaking incubator (Thermo Scientific, Waltham, MA, USA). Subsequently, PHB production was evaluated in a 500 mL Erlenmeyer flask containing 200 mL of GRPD growth medium. This was inoculated with 2% (*v*/*v*) of 24 h-old pre-inoculum and incubated at 150 rpm and 30 °C for 48 h using a MaxQ4000 shaking incubator (Thermo Scientific, Waltham, MA, USA).

#### 2.4.2. PHB Extraction Protocol

PHB extraction and purification were carried out following the protocol reported by Martínez-Herrera et al. [20]. In the first stage, cellular biomass was collected after fermentation by centrifugation at 4 °C for 15 min at 10,000× *g* using a J251 ultracentrifuge (Beckman Coulter, Brea, CA, USA), and the supernatant was discarded. In the second stage, the obtained pellets were treated with commercial NaOCl (Cloralex^®^, Oakland, CA, USA) and incubated for 30 min at 20 °C in a 5510R-MT ultrasound bath (Branson, CT, USA). The digested sample was then centrifuged, and the pellets were washed with distilled water and centrifuged. In the third stage, the resulting pellets were treated with chloroform and boiled in a water bath for 1 min. The extract was placed in pre-weighed glass Petri dishes and dried overnight.

#### 2.4.3. Quantification of Biomass Generation

The assessment of biomass generation followed the methodology outlined by Aramvash et al. [21]. To do this, 10 mL of growth medium was dispensed into pre-weighed 2 mL microtubes and centrifuged for 15 min at 1900× *g* using a TM22R microcentrifuge (Beckman Coulter, Brea, CA, USA). After discarding the supernatant, the pellets were washed with distilled water and subsequently dried at 60 °C for 24 h; finally, the microtubes were weighed using an AG204 analytical balance, with an accuracy of 0.1 mg (Mettler Toledo, Columbus, OH, USA). The total biomass generation was quantified in grams per liter (g/L).

#### 2.4.4. Quantification of PHB Accumulation Percentage

The PHB accumulation percentage (%PHB) was calculated employing the following formula [22]:

$$\%PHB=\left[\frac{P}{X}\right]\times 100$$

where P is the PHB production calculated in g/L and X is the biomass generation calculated in g/L.

### 2.5. Statistical Analysis

The quantitative analysis (see Section 2.4) was performed in triplicate. Likewise, these data were statistically compared and evaluated by employing a One-Way Analysis of Variance (ANOVA), as well as a Tukey posterior test for homogeneous groups; a *p*-value ≤ 0.05 was considered significant. Data are expressed as mean values ± standard deviation. The parameters evaluated were biomass generation (g/L) and PHB production (g/L). Statistical analysis was performed using SPSS v20 software.

### 2.6. Biochemical and Molecular Characterization

#### 2.6.1. Biochemical Characterization

The identification of the selected bacterial strains (both qualitatively and quantitatively) was carried out through macroscopic and microscopic examinations, as well as biochemical tests. Macroscopic identification involved assessing colony morphology, surface pigment, shape, and size on nutrient agar plates. Microscopic examination included Gram staining to study staining behavior, cell arrangement and granulation, along with spore staining. Furthermore, a battery of biochemical tests was conducted, including the oxidase test, catalase test, Sulfur, Indole, and Motility (SIM) test, citrate test, Triple Sugar Iron (TSI) test, Lysine Iron Agar (LIA) test, Motility Indole Ornithine (MIO) test, urea test, Methyl Red test, Voges–Proskauer (MR-VP) test, Growth on NaCl 6.5% test, starch hydrolysis test, and lecithinase test.

#### 2.6.2. Molecular Characterization

##### DNA Extraction

Genomic DNA extraction was conducted from 24 h liquid culture, isolates using the modified phenol–chloroform method [23]. Initially, to prepare the cell wall of the isolate, multiple washes with TE Buffer were performed. Subsequently, the pellet from these washes was resuspended in lysis buffer, and 0.1 mm glass beads were added. Using a MagnaLyser instrument, four cycles of 40 s, with 1 min rest intervals, were applied. Using the supernatant, the subsequent steps involved phenol–chloroform washes to separate the genetic material from impurities, followed by 70% ethanol washes to remove residual phenol. Finally, using the precipitate, it was quantified using a nanodrop spectrophotometer for quality and concentration assessment. Agarose gels were then prepared for electrophoresis, running the DNA samples with GelRed and the Hyperleadeff 1 kb marker. The agarose gel was set at a 1% concentration, and the samples were electrophoresed at 110 V for 45 min. Subsequently, the gels were visualized using a UV transilluminator.

##### Phylogenetic Analysis by 16S rRNA Sequencing

As part of the polymerase chain reaction (PCR) methodology, universal primers 27F (5′-AGAGTTTGATCMTGGCTCAG-3′) and 1492R (5′-CGGTTACCTTGTTACGACTT-3′) were subjected to amplification in a reaction volume of 15 µL. This composition included 0.5 µg of the isolated genomic DNA, 0.5 µL for each primer, 7.5 µL of DreamTaq PCR master mix (2X) (Thermo Fisher Scientific), and 6 µL of nuclease-free water. The thermal cycler (Eppendorf, Hamburg, Germany) utilized for the PCR-cycling protocol was defined as follows: an initial denaturation at 95 °C for 3 min, 30 cycles of denaturation at 95 °C for 30 s, primer annealing at 49 °C for 30 s, and extension at 72 °C for 1 min, followed by a final extension step at 72 °C for 10 min. Subsequently, the amplified PCR products underwent re-examination through electrophoresis on a 1% agarose gel.

The PCR product was cloned using the pGEM t easy vector system (Promega, Tokyo, Japan), according to the manufacturer’s instructions and following the method published by Fuentes et al. [24]. Finally, the genomic DNA sample was sent to be sequenced by Sanger technology for the 16S rRNA gene using a 3130 genetic analyzer (Applied Biosystems, Waltham, MA, USA). The sequence was derived from the resulting electropherograms, and the isolate was identified using the BLAST tool (https://blast.ncbi.nlm.nih.gov/Blast.cgi, accessed on 15 December 2023).

The 16S ribosomal sequence, obtained from the primers, was processed in Bioedit v 7.0.5.3. The sequence was subsequently queried against the NCBI database (https://blast.ncbi.nlm.nih.gov/Blast.cgi?PROGRAM=blastn&PAGE_TYPE=BlastSearch&LINK_LOC=blasthome, accessed on 15 December 2023) using the BLAST tool to identify the closest matches to the isolate sequences. Subsequently, the sequence was aligned, incorporating reference sequences obtained from the NCBI database. The alignment comprised 15 sequences and was further processed in MEGA11. Next, this alignment was analyzed to determine the optimal substitution model for constructing the phylogenetic tree (in this case, the Kimura 2-parameter). Finally, within the same software, the phylogenetic tree was constructed using the “Maximum Likelihood”.

### 2.7. Optimization of PHB Production and Kinetic Studies

#### 2.7.1. Optimization of Incubation Parameters

To determine the optimal parameters for PHB production, a Design of Experiments (DoE) methodology was employed using the Taguchi experimental design, which aims to optimize individual yield characteristics [25]. This design utilized a loss function to measure the deviation between the experimental values and the desired values. This loss function is then transformed into a signal-to-noise (S/N) ratio. In the analysis of the S/N ratio, three types of quality characteristics are considered: ‘the smaller the better’, ‘the bigger the better’, and ‘the nominal the best’ [26]. In this study, ‘the nominal the best’ approach was employed for the experimentation and validation of PHB optimization processes [25]. This approach included four factors with five levels each: pH (6, 6.5, 7, 7.5, and 8), temperature (28, 30, 32, 34, and 36 °C), inoculum size (1, 2, 3, 4, and 5% *v*/*v*), and agitation speed (110, 120, 130, 140, and 150 rpm), which were identified and selected for PHB optimization (Table 1). Next, an experimental matrix was created to account for variations in the control factors at the five different levels. A Taguchi orthogonal array design L25 (5*4) was used for conducting 25 experimental trials to determine and analyze the optimal PHB biosynthesis processes. The validation analysis was based on the designed experiments and their results, using the signal-to-noise (S/N) ratio. During this step, biomass generation (g/L), PHB (g/L), and %PHB were considered for validation. The obtained data was analyzed using Minitab 17v software.

#### 2.7.2. Analysis of Bacterial Growth Kinetics

Growth kinetics analysis of the selected bacterial strain under optimal conditions was conducted in triplicate. This kinetic analysis was performed at 0, 6, 12, 18, 24, 36, 48, 60, and 72 h, while varying glucose concentrations (0, 5, 10, 15, 20, and 25 g/L) were used to determine the optimal concentration; it used a One-Way Analysis of Variance (ANOVA). Additionally, the 3,5-dinitrosalicylic acid (DNS) method was employed to measure sugar concentrations throughout the fermentation time [27]. From the experimental data, the following kinetic parameters were calculated to evaluate growth and PHB production behavior [28,29,30].

$$µ=\frac{ln\left(\frac{X}{X_{0}}\right)}{T-T_{0}}$$

where µ is the specific growth rate (h^−1^) and X_2_ and X_1_ are the biomass generation (g/L) obtained at time T_1_ and T_2_, respectively.

$$Td=\frac{LN(2)}{µ}$$

where Td represents the doubling time (h), which is calculated as the natural logarithm of 2 divided by µ (h^−1^).

$$Y_{P/X}=\frac{\left(P_{2}-P_{1}\right)}{\left(X_{2}-X_{1}\right)}$$

$$Y_{X/S}=\frac{\left(X_{2}-X_{1}\right)}{\left(S_{1}-S_{2}\right)}$$

$$Y_{P/S}=\frac{\left(P_{2}-P_{1}\right)}{\left(S_{1}-S_{2}\right)}$$

where Y_P/X_ represents the product yield on biomass (g/g), calculated as the difference between the PHB produced (g/L) and the biomass generated (g/L). Y_X/S_ represents the biomass yield on substrate (g/g), calculated as the difference between the biomass generated (g/L) and the substrate consumed (g/L). Y_P/S_ represents the product yield on substrate (g/g), calculated as the difference between the PHB produced (g/L) and the substrate consumed (g/L).

$$\mathrm{Qp}=\frac{P}{\left(T_{2}-T_{1}\right)}$$

where Qp is the productivity rate (g/L*h), which is calculated as the ratio between the PHB produced (g/L) and the fermentation time (h).

### 2.8. PHB Characterization

#### 2.8.1. Fourier-Transform Infrared Spectroscopy (FTIR)

The characterization of functional groups was analyzed using Fourier-transform infrared (FTIR) spectra, which were obtained with a PerkinElmer Spectrum Frontier spectrometer. A piece of biopolymer was scanned in the range of 400–4000 cm^−1^, with 25 scans recorded per sample, using a resolution of 4 cm^−1^ and the ATR mode. Analysis of the spectra was conducted using GraphPad Prism 8 software.

#### 2.8.2. Differential Scanning Calorimetry (DSC)

The thermal transitions of the composites were determined by DSC using a Q2000 differential scanning calorimeter (TA Instruments, New Castle, DE, USA). Samples were placed into an aluminum hermetic pan, which was sealed and scanned over a range from 25 to 300 °C at a heating rate of 5 °C/min. Thermal properties, including melting enthalpy (ΔHm), melting temperature (Tm), crystallization temperature (Tc), and degradation temperature (Td), were computed from the thermograms using Universal Analysis 2000 software v 2.0. All experiments were conducted in triplicate. In all cases, the sealed pans that contained samples were equilibrated at 25 °C for 1 h before DSC analysis. The crystallinity (χ) was evaluated using the formula reported by S. Maity et al. [31].

Χc(%)=(ΔHm)/(ΔHmo)×100

where ΔHm is the melting enthalpy of the sample and ΔHmo is the heat of fusion of standard PHB (146 J/g).

## 3. Results and Discussions

### 3.1. Isolation and Qualitative Screening

**Figure 1:**
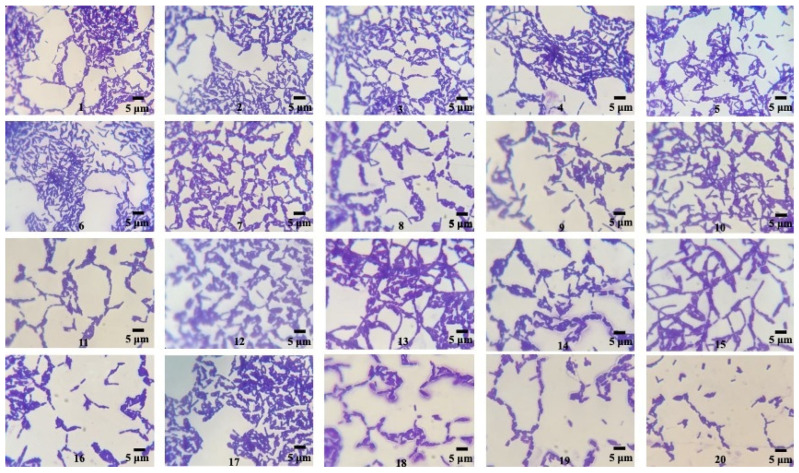
Microscopic morphology of the isolated *Bacillus* strains (1–20) observed through optical microscopy after 24 h, 30 °C, Nutritive agar, Gram staining (100×).

From the soil sample collected in our study, twenty bacteria were isolated with different colony morphologies, obtained by pure culture techniques in nutritive agar media. The isolate strains presented macroscopic morphological characteristics (circular colonies of medium size exhibiting a grayish-white hue, opacity, and a flat, drying morphology) and microscopic characteristics (Gram-positive, endospore-forming, bacilli shape) typical of the genus *Bacillus* [32] (Figure 1). These bacterial strains were utilized for evaluation in a qualitative screening of PHB producers.

**Figure 2:**
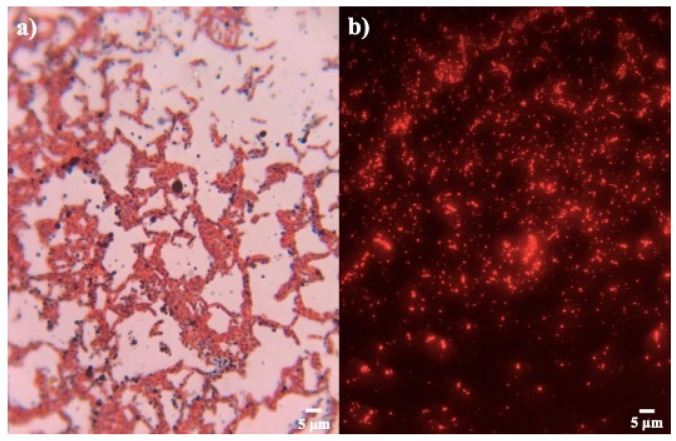
Staining for confirmation PHB production. (**a**) Sudan black B and (**b**) Nile blue A of strain *Bacillus* sp. 12GS (300 ppp).

The strains proved to be positive in the production of PHB. Through Sudan Black B and safranin staining, granules composed of fatty acids, colored black, were observed inside pink cytoplasm (Figure 2a). This stain is useful, because the size and volume that the granule occupies within the cell can be observed, and it is considered a specific stain in the search for strains that produce this biopolymer [33,34].

*Bacillus* strains were also tested with Nile blue A for confirmation PHB production (Figure 2b). It was observed that the isolates presented a different biopolymer production, due to the presence of various degrees of orange-red fluorescence in all the strains, using fluorescence microscopy [35]. This staining allows us to confirm and select the bacterial strains in PHB producers by the intensity of fluorescence (Table 2). Bhagowati et al. [35] considered an integrated density value of 6513.5 as a sign of a high producer of PHB. In this way, strains of our study with the highest reported values, according to the results of Sudan Black B, were selected for quantitative screening. The strains B7, B8, B10, B15, B16, and B19 were discarded for low fluorescence.

### 3.2. Quantitative Screening of the Bacterial Isolates

The selected isolates (B1, B2, B3, B4, B5, B6, B9, B11, B12, B13, B14, B17, B18, and B20) were evaluated for quantitative screening. The results show that all the tested isolates produced PHB (Table 3). The dry biomass ranged from 0.96 to 4.6 g/L; these results were like the studies by Amiri et al. [36], which obtained a maximum 4.3 g/L of biomass using *Bacillus* strains isolated from petrochemical wastewater, and Martínez-Herrera et al. [20], which obtained 3.76 g/L using GRPD media and *Bacillus* isolated from soil. Likewise, Thammasittirong et al. [37] obtained lower values of twenty isolates of *Bacillus* using 1% of sucrose. PHB production varied from 0.31 to 0.65 g/L. All the strains were higher than those found by Yadav et al. [38]; they obtained 0.07 g/L using a strain of *Bacillus subtilis,* and Sun et al. [39] obtained 0.55 g/L using *Bacillus* sp. Other studies have reported findings similar to this work in PHB g/L [40]. Likewise, the %PHB ranged between 18 and 40.7%; this yield was higher than other investigations reported by El-Kadi et al. [41] where they obtained 20% PHB, using glucose as a carbon source. Other studies obtained values with a similar %PHB to our work [42]. The strain B12 produced the highest dry biomass production of 4.6 g/L, along with a PHB g/L production of 0.65 g/L.

A significant difference (*p* ≤ 0.05) was observed between bacterial isolates only for biomass g/L production. Therefore, the best isolate that yielded the highest biomass was B12; this isolate was chosen and used in the subsequent experiments, with the intention of increasing biopolymer production with the experimental design.

### 3.3. Biochemical and Molecular Characterization

The strain of *Bacillus* selected (B12 or *Bacillus* sp. 12GS) is Gram-positive and forms endospores after Shaffer Fulton and Gram techniques staining; the catalase and oxidase test were positive, and likewise, the lecithinase and starch hydrolysis were positive. These positive biochemical tests, characteristics of *Bacillus cereus*, agree with Amiri et al. and Pirttijärvi et al. [43,44]. Table 4 summarizes the biochemical and morphological characteristics of the strain.

**Figure 3:**
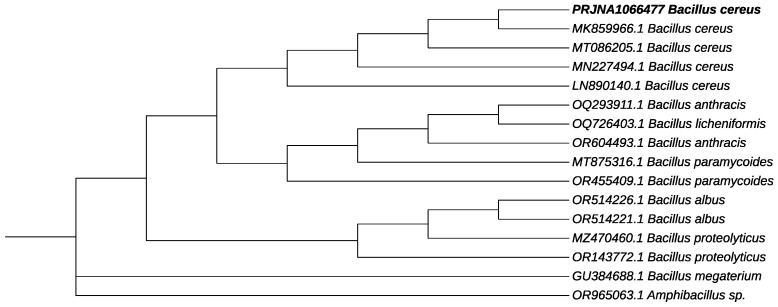
Phylogenetic tree on the genetic relationship of PRJNA1066477 *B. cereus* 12GS (showed in bold). The accession number in GenBank is presented for each strain. *Amphibacillus* sp. was used as an outgroup.

In order to amplify the 16S rRNA gene of B12 isolate, genomic DNA was extracted and used for PCR amplification. As a result of the 16S rRNA gene amplification and sequencing, the B12 strain was found to share a high identity with *Bacillus cereus*. The sequence of the gene that was obtained has been deposited with GenBank with the accession number PRJNA1066477 and is referred to as *Bacillus cereus* 12GS. Phylogenetic trees were constructed for the strain and other *Bacillus* species (Figure 3). Previous researchers have conducted similar studies involving the analysis of the 16S rRNA gene for the identification of unknown bacterial isolates that produce PHB. New strains of PHB-producing *B. cereus* have been isolated from different ecosystems and were identified by analyzing 16S rRNA sequences, including bacteria from TNT-contaminated soil (*B. cereus* FA11) [42], petrochemical wastewater (*B. cereus* saba.zh) [43], plastic waste (*B. cereus* BNPI-92) [25], and soil (*B. cereus* VIT-SSR1) [33], (*B. cereus* 4N) [20].

*B. cereus* strains can produce these biopolymers under various nutritional and incubation conditions, such as an excess of carbon sources and nitrogen limitations [45]. Several growth media have been identified for PHA production with *B. cereus*, and the type of substrate added to the growth media does not impede its ability to produce PHAs. In general, *B. cereus* strains can thrive and produce PHAs under conditions of uncontrolled pH, moderate temperatures, and agitation speeds ranging from 120 to 250 rpm [46]. These conditions should ensure optimal levels of dissolved oxygen, efficient mass transfer, and prevent biopolymer degradation [19,47]. Variations in nutritional and incubation parameters, including pH, temperature, agitation speed, and fermentation time, affect PHA productivity and the physicochemical properties of the extracted biopolymer. This variability is attributed to metabolic plasticity, as optimal parameters and productive behaviors differ among *B. cereus* strains and are often strain-specific. Indeed, each strain tends to respond according to its original ecological niche. Frequent fluctuations in organic matter and chemical compounds are common and influence the genetic and metabolic properties of these strains, affecting their adaptability and productivity [46].

### 3.4. Optimization of PHB Production and Kinetic Studies

**Figure 4:**
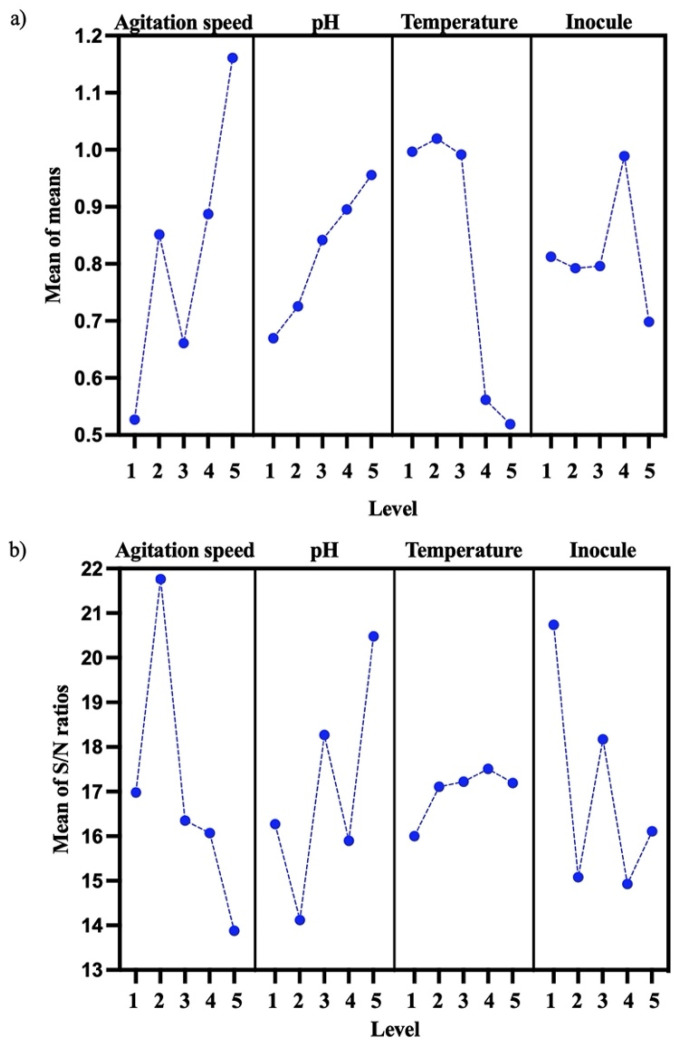
Effect of control factor (agitation speed, pH, temperature, and inocule) for PHB optimization. (**a**) Mean of control factor for PHB (g/L). (**b**) Mean of S/N ratio for control factor on PHB (g/L).

PHB biosynthesis was optimized for the *B. cereus* 12GS strain using the DOE Taguchi method (Table 5). Levels, means, and S/N ratios for the control factor that were able to give the best value of PHB concentration (g/L) were calculated for factor a (level 5, mean = 1.1613 and S/N = −13.88), factor b (level 5, mean = 0.9577 and S/N = 20.48), factor c (level 2, mean = 1.0195, and S/N = 17.11), and factor d (level 4, mean = 0.9889, and S/N = 14.93) (Figure 4a). An optimum PHB (g/L) mean value was predicted for 150 rpm, pH 8, 30 °C, and 4% inoculum, as shown in Figure 4 (the best level for each factor). The most important parameters were the agitation speed and pH. According to other studies, Masood et al. [19] previously reported 150 rpm as the best agitation speed for PHB (g/L) production using *B. cereus* FA11, and Mascarenhas et al. [48] reported pH 8 as the best for PHB production using *B. megaterium* JHA.

This suggests that the experimental design used to optimize PHB production was based on the combination of different factors and their individual performance [25,49]. These parameters (150 rpm, pH 8, 30 °C, and 4% inoculum) were probed, and the results of the optimization showed an increased PHB production, the values increasing by 0.65 g/L PHB and 14.2% PHB in the quantitative screening (Section 3.4) to 1.91 g/L PHB and 87.2% PHB (Table 6). Ronďošová et al. [50] optimized the medium for PHB production and obtained 49% PHB. As well, García et al. [51] report 29% PHB using the Taguchi experimental design. By evaluating the signal-to-noise (SN) ratio, it becomes possible to discern favorable characteristics (Figure 4b). These discrepancies could be because *Bacillus* strains demonstrate a variety of productive behaviors that are specific to each strain. In fact, each strain tends to respond according to its original place of isolation [46]. The parameter exhibiting the greater SN ratio signifies the highest impact factor. Consequently, this approach facilitates the optimization of the parameters. The experimental design detailed the input factors and their associated responses [52]. In this case, the highest SN ratio is factor a (agitation speed), then factor 3 (pH), factor 4 (inocule), and finally, factor 2 (temperature).

**Figure 5:**
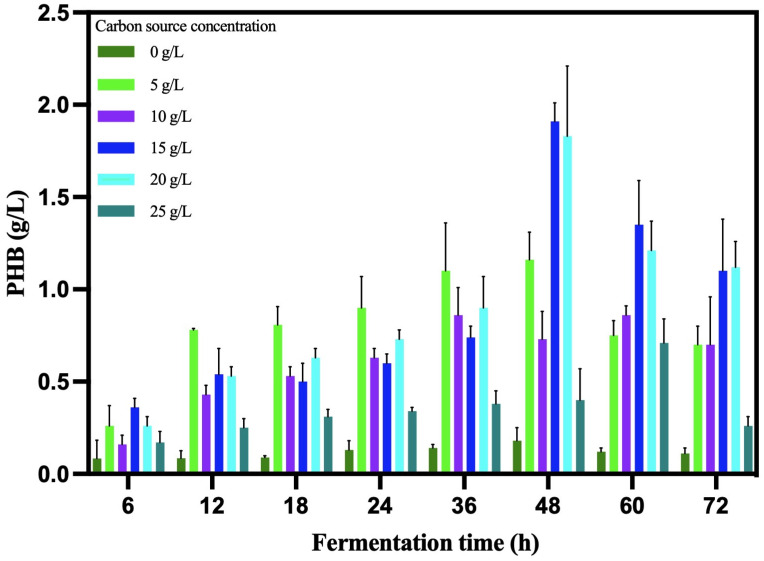
Kinetic analysis of PHB production by *B. cereus* 12GS.

The kinetic analysis showed the best concentration at 20 g/L (Figure 5). The *B. cereus* 12GS strain showed a 0.07 h^−1^ specific growth rate with a doubling time of 9.90 h (Table 7). Likewise, the PHB biomass yield value (Y p/x) was 0.52 g/L*h. Other strains of *Bacillus* reported lower values, around 0.40 g/L*h [53,54]. The PHB volumetric productivity value (Qp) was 0.04 g/L*h, a value higher than reported by Ahmady-Asbchin et al. [55] and Patel et al. [56] using glucose as a carbon source. On the other hand, other *Bacillus cereus* were reported to have similar and lower values, utilizing 30 °C and 150 rpm in liquid fermentation [57,58,59]. The time at which *B. cereus* 12GS reaches a maximum PHB biosynthesis (48 h) is one of the advantages for the competitive production of this biopolymer at the industrial level [60,61].

### 3.5. PHB Characterization

#### 3.5.1. Fourier-Transform Infrared Spectroscopy (FTIR)

**Figure 6:**
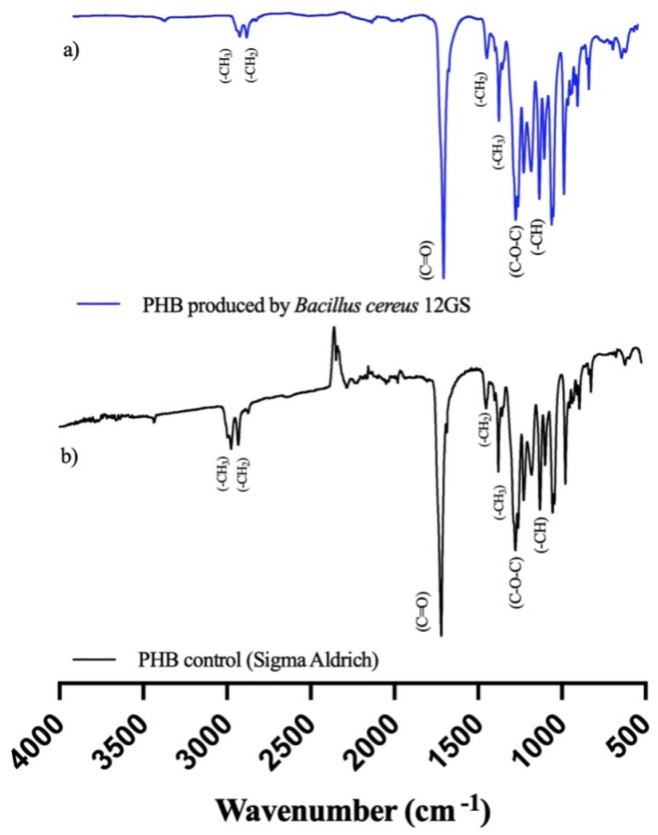
Fourier-Transform Infrared (FTIR) spectroscopy analysis of (**a**) PHB produced by *B. cereus* 12GS, (**b**) commercial PHB (Sigma-Aldrich).

Fourier-Transform Infrared (FTIR) spectroscopy analysis is a tool to show the chemical functional groups present in the examined extract. The spectral pattern encompassing a range from 400 to 4000 cm^−1^ is graphically shown in Figure 6. Notably, the formed peaks at approximately 1719 cm^−1^ are attributable to the carbonyl group (C=O) characterizing the ester. An additional peak at 1276 cm^−1^ is suggestive of the (-CH) group. Further examination reveals a distinctive band appearing at 1450 cm^−1^, associated with the asymmetric deformation of the (C-H) bonds within both the (-CH_2_) and (-CH_3_) groups, with another notable occurrence at 1379 cm^−1^. Within the range of 1000 to 1300 cm^−1^, a series of pronounced bands signifies the stretching of the (C-O) bond inherent to the ester group. Importantly, all these spectral features align closely with the standard commercial polyhydroxybutirate (Sigma-Aldrich) (Figure 6); therefore, it is confirmed that the polymeric extract studied is a short chain named polyhydroxybutyrate (PHB) [29,62].

#### 3.5.2. Differential Scanning Calorimetry (DSC)

**Figure 7:**
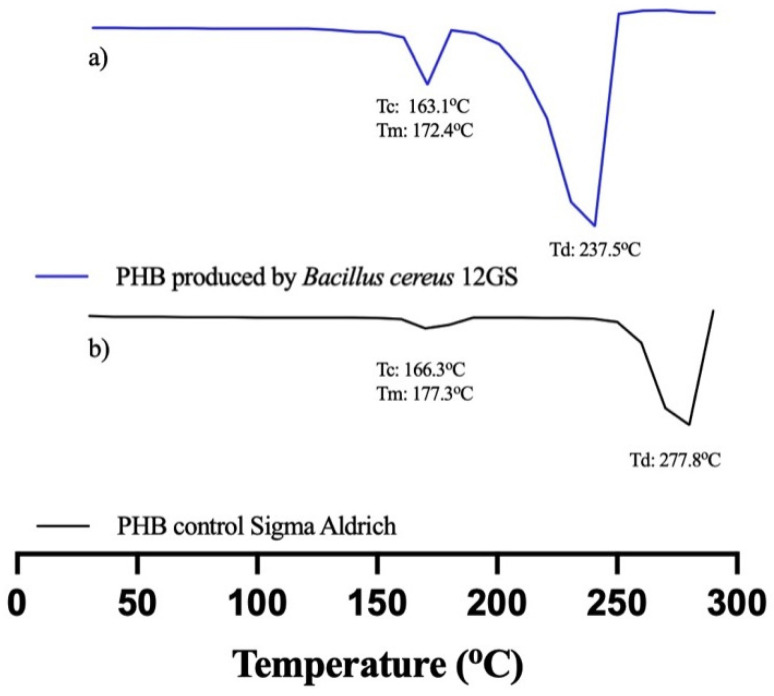
Differential scanning calorimetry (DSC) of (**a**) PHB produced by *B. cereus* 12GS, (**b**) commercial PHB (Sigma-Aldrich).

*B. cereus* 12GS has been found to be a potent producer of polyhydroxybutyrate (PHB), a biodegradable plastic characterized by promising thermal characteristics. Analysis of the thermal properties of PHB was performed using DSC (Table 8), conduced to investigate the melting enthalpy (ΔHm), melting temperature (Tm), crystallization temperature (Tc), degradation temperature (Td), and crystallinity (χ) of PHB. The Tm and crystallinity of PHB were found to be 172.49 °C and 42%, respectively; other studies [63] reported a similar-value Tm of 165.6 °C for PHB produced by *Bacillus megaterium*. Bhagowati et al. [35] reported a melting temperature (Tm) of 109.4 °C for PHB synthesized by this bacterium. This observation was corroborated by Rehman et al. [64], who noted a Tm of 162 °C and a crystallinity of 51.3%. However, Pati et al. [65] reported a lower Tm of 171 °C and a crystallinity of 35%. The Tc found was 163.17 °C, a higher value compared to Martínez et al. [20], using glucose as a carbon source in PHB production. The Td was found to be 237.24 °C, a similar value to what was reported in the literature [63,65]. The ΔHm was reported as 61.55 J/g, a result similar to Oliveira et al. [66], with 66.9 J/g for PHB produced by fermentation with *Cupriavidus necator*. The thermogram was compared with standard PHB (Sigma-Aldrich) (Figure 7). The variations observed stem from disparities in the production and extraction methodologies, as well as the bacterial strain used. Despite these discrepancies, the thermal characteristics of polyhydroxybutyrate (PHB) synthesized by *B. cereus* 12GS generally exhibit favorable attributes, making it a promising candidate for diverse applications; likewise, the high thermal stability of polyhydroxyalkanoates (PHAs) represents an important parameter in the polymerization process, as it permits the polymer to reach elevated temperatures [67]. The results obtained by DSC indicate that the PHB produced by *B. cereus* 12GS exhibits a lower temperature and molecular weight (apparently), compared to the control PHB. However, this positions it as a polymer of low fragility but with a better biodegradation rate, making it suitable for applications in disposable articles and food packaging [68,69,70].

## 4. Conclusions

This study demonstrates a strain of *B. cereus* recently isolated from agricultural soil as a high PHB producer up to 87%, without specific growth requirements in the culture medium. Moreover, when exposed to alkaline pH, it exhibits an enhanced yield, which is unusual for a bacterial strain of this genus. This was evidenced through the Taguchi Design of Experiments (DoE), which facilitates the identification of the most favorable production conditions. Additionally, chemical analyses were crucial in confirming the structure of short-chain-length polyhydroxyalkanoate (scl-PHA), denominated as polyhydroxybutyrate (PHB).

## Figures and Tables

**Table 1:** Factors and levels used in Taguchi experimental design to produce PHB.

Parameter	Factor	Level				
		1	2	3	4	5
Agitation speed (rpm)	A	110	120	130	140	150
pH	B	6	6.5	7	7.5	8
Temperature (°C)	C	28	30	32	34	36
Inoculum (%)	D	1	2	3	4	5

**Table 2:** Qualitative evaluation of PHB by *Bacillus* strains.

Stainings of PHB (Qualitative Screening)		
Strain	Sudan Black B (Production)	Nile Blue A (Integrated Density)
B1	(++)	44,013.228
B2	(+++)	166,705.709
B3	(+++)	728,499.860
B4	(++)	31,617.513
B5	(++)	87,029.191
B6	(++)	8140.101
B7	(+)	2472.227
B8	(+)	2551.696
B9	(++)	7311.102
B10	(+)	4247.510
B11	(++)	173,663.663
B12	(+++)	1,695,242.386
B13	(++)	123,182.626
B14	(++)	72,675.122
B15	(+)	5060.914
B16	(+)	4856.413
B17	(+++)	175,356.035
B18	(+++)	347,629.345
B19	(+)	5420.557
B20	(++)	29,617.563

(+): Low production, (++): Medium production, (+++): High production.

**Table 3:** Production of biomass g/L, PHB g/L, and %PHB by *Bacillus* strains.

Strain	Biomass g/L	PHB g/L	% PHB
B1	0.96 ± 0.18 ^e^	0.39 ± 0.08 ^a^	40.7 ^a^
B2	1.67 ± 0.3 ^b,c,d,e^	0.43 ± 0.15 ^a^	25.8 ^a^
B3	1.49 ± 0.07 ^d,e^	0.59 ± 0.18 ^a^	39.8 ^a^
B4	1.4 ± 0.1 ^d,e^	0.39 ± 0.14 ^a^	28.1 ^a^
B5	1.84 ± 0.22 ^b,c,d,e^	0.52 ± 0.1 ^a^	29.8 ^a^
B6	1.73 ± 0.11 ^b,c,d,e^	0.31 ± 0.05 ^a^	18 ^a^
B9	1.88 ± 0.07 ^b,c,d,e^	0.48 ± 0.14 ^a^	25.7 ^a^
B11	1.95 ± 0.26 ^b,c,d,e^	0.41 ± 0.01 ^a^	21.1 ^a^
B12	4.6 ± 0.7 ^a^	0.65 ± 0.15 ^a^	14.2 ^a^
B13	2.81 ± 0.5 ^b^	0.49 ± 0.16 ^a^	17.3 ^a^
B14	2.48 ± 0.27 ^b,c,d^	0.44 ± 0.09 ^a^	17.6 ^a^
B17	2.69 ± 0.9 ^b,c^	0.63 ± 0.15 ^a^	18 ^a^
B18	2.42 ± 0.17 ^b,c,d^	0.44 ± 0.14 ^a^	18.3 ^a^
B20	1.63 ± 0.08 ^c,d,e^	0.64 ± 0.15 ^a^	39.1 ^a^

Note: Superscript letters (a–e) indicate significant differences between treatments.

**Table 4:** Biochemical and morphological characteristics of *Bacillus* sp. 12GS and *Bacillus cereus* saba.zh.

Characterization	*Bacillus* sp. 12GS	*Bacillus cereus* saba.zh [43]
Shape	Rod	Rod
Gram staining	Gram-positive (+)	Gram-positive (+)
Sporulation	(+)	(+)
Oxidase	(+)	(+)
Catalase	(+)	(+)
Sulfur	(−)	—
Indol	(−)	(−)
Motility	(−)	—
Citrate	(−)	(+)
Triple Sugar Iron	(K/A)	(K/A)
Lysine Iron Agar	(−)	—
Motility Indole Ornithine	(−)	—
Urea	(+)	(−)
Methyl Red	(−)	(−)
Voges–Proskauer	(−)	(+)
Growth on NaCl 6.5%	(−)	—
Starch hydrolysis	(+)	(+)
Lecthinase	(+)	—

(+): Positive reaction, (−): Negative reaction, (K/A): Alkaline/Acid. —: Not mentioned.

**Table 5:** Experimental design using orthogonal array of Taguchi L25 (5*4).

Experiment No.	Agitation Speed (rpm)	pH	Temperature (°C)	Inocule (%)	Biomass (g/L)	PHB (g/L)	% PHB
1	110	6	28	1	1.89 ± 0.57	0.65 ± 0.12	52.24
2	110	6.5	30	2	1.86 ± 0.14	0.58 ± 0.21	31.35
3	110	7	32	3	1.93 ± 0.52	0.62 ± 0.13	31.92
4	110	7.5	34	4	1.7 ± 0.01	0.53 ± 0.15	30.9
5	110	8	36	5	1.33 ± 0.07	0.25 ± 0.12	19.19
6	120	6	30	3	1.82 ± 0.11	1.01 ± 0.05	55.45
7	120	6.5	32	4	2.29 ± 0.18	0.93 ± 0.2	40.37
8	120	7	34	5	1.96 ± 0.17	0.58 ± 0.04	29.61
9	120	7.5	36	1	2.06 ± 0.05	0.61 ± 0.09	29.52
10	120	8	28	2	2.6 ± 0.06	1.14 ± 0.07	43.76
11	130	6	32	5	2.16 ± 0.12	0.63 ± 0.22	32.33
12	130	6.5	34	1	2.03 ± 0.27	0.42 ± 0.11	20.5
13	130	7	36	2	1.45 ± 0.08	0.3 ± 0.14	20.48
14	130	7.5	28	3	2.27 ± 0.11	0.7 ± 0.14	30.77
15	130	8	30	4	2.52 ± 0.34	1.26 ± 0.15	50.03
16	140	6	34	2	2.02 ± 0.41	0.53 ± 0.1	26.18
17	140	6.5	36	3	2.28 ± 0.25	0.55 ± 0.4	42.07
18	140	7	28	4	2.61 ± 0.25	1.52 ± 0.13	58.35
19	140	7.5	30	5	2.96 ± 0.43	0.97 ± 0.22	32.75
20	140	8	32	1	3.26 ± 0.32	1.11 ± 0.04	34.09
21	150	6	34	4	3.42 ± 0.09	0.79 ± 0.19	23.10
22	150	6.5	28	5	4.33 ± 0.62	1.06 ± 0.33	24.46
23	150	7	30	1	2.6 ± 0.17	1.28 ± 0.14	48.78
24	150	7.5	32	2	2.95 ± 0.12	1.67 ± 0.23	56.60
25	150	8	34	3	2.2 ± 0.21	1.01 ± 0.16	45.98

**Table 6:** Comparison of *B. cereus* 12GS production under original and optimized conditions.

Conditions	Biomass (g/L)	PHB (g/L)	% PHB
Original (pH 6, 30 °C, 2% inocule, 150 rpm)	4.6 ± 0.7	0.65 ± 0.15	14.2
Optimized (pH 8, 30 °C, 4% inocule, 150 rpm)	2.19 ± 0.05	1.91 ± 0.1	87.2

**Table 7:** Kinetic parameters calculated for *B. cereus* 12GS.

Kinetic Parameter	Value
µ (h^−1^)	0.07
µmax (h^−1^)	0.09
Td (h)	9.90
Ks (g/L)	5
Y p/x (g/L*h)	0.52
Y x/s (g/L*h)	0.22
Y p/s (g/L*h)	0.15
Y x + p/s (g/L*h)	0.37
qs (g/L*h)	0.09
qp (g/L*h)	0.014
Qp (g/L*h)	0.04

**Table 8:** Thermal properties of the commercial PHB control (Sigma-Aldrich) and the PHB produced by *B. cereus* 12GS.

Sample	Tm (°C)	ΔHm (J/g)	Tc (°C)	Td (°C)	Xc (%)
PHB control (Sigma-Aldrich)	177.38	76.78	166.33	277.83	52.59
PHB by *B. cereus* 12GS	172.49	61.55	163.17	237.82	42

## Data Availability

Data are contained within the article.
